# Non-Fungible Tokens (NFTs) in Healthcare: A Systematic Review

**DOI:** 10.3390/ijerph21080965

**Published:** 2024-07-24

**Authors:** Tiago Nunes, Paulo Rupino da Cunha, João Mendes de Abreu, Joana Duarte, Ana Corte-Real

**Affiliations:** 1 Faculty of Medicine, University of Coimbra, 3000-548 Coimbra, Portugal; 2 Centre for Informatics and Systems of the University of Coimbra (CISUC), Department of Informatics Engineering, University of Coimbra, 3030-790 Coimbra, Portugal; 3 Clinical and Academic Centre of Coimbra, 3004-561 Coimbra, Portugal; 4 Associated Laboratory for Energy, Transports and Aeronautics (LAETA-PROA), Faculty of Engineering, University of Porto, 4200-465 Porto, Portugal

**Keywords:** blockchain, non-fungible tokens, digital health, medical informatics, electronic health records

## Abstract

Amid global health challenges, resilient health systems require continuous innovation and progress. Stakeholders highlight the critical role of digital technologies in accelerating this progress. However, the digital health field faces significant challenges, including the sensitivity of health data, the absence of evidence-based standards, data governance issues, and a lack of evidence on the impact of digital health strategies. Overcoming these challenges is crucial to unlocking the full potential of digital health innovations in enhancing healthcare delivery and outcomes. Prioritizing security and privacy is essential in developing digital health solutions that are transparent, accessible, and effective. Non-fungible tokens (NFTs) have gained widespread attention, including in healthcare, offering innovative solutions and addressing challenges through blockchain technology. This paper addresses the gap in systematic-level studies on NFT applications in healthcare, aiming to comprehensively analyze use cases and associated research challenges. The search included primary studies published between 2014 and November 2023, searching in a balanced set of databases compiling articles from different fields. A review was conducted following the Preferred Reporting Items for Systematic Reviews and Meta-Analyses (PRISMA) framework and strictly focusing on research articles related to NFT applications in the healthcare sector. The electronic search retrieved 1902 articles, ultimately resulting in 15 articles for data extraction. These articles span applications of NFTs in medical devices, pathology exams, diagnosis, pharmaceuticals, and other healthcare domains, highlighting their potential to eliminate centralized trust sources in health informatics. The review emphasizes the adaptability and versatility of NFT-based solutions, indicating their broader applicability across various healthcare stages and expansion into diverse industries. Given their role in addressing challenges associated with enhancing data integrity, availability, non-repudiation, and authentication, NFTs remain a promising avenue for future research within digital health solutions.

## 1. Introduction

In the face of the recent global health threats, pandemics, armed conflicts, increased service demands, and an aging population, significant challenges confront the resilience of health systems [1]. The increasing demand for healthcare systems to address these challenges and be able to prevent, prepare for, detect, and recover from them underscores the need for ongoing innovation and progress in the health sector [1].

The spread of information, communication, and global interconnectedness using digital technologies has been pointed out in the 2030 Agenda for Sustainable Development as showing great potential to address the digital divide. Hence, the World Health Organization (WHO) advocates using digital technologies to accelerate the global attainment of health and well-being [2].

Digital technology innovation is happening on an unprecedented scale. However, its use for digital health solutions remains to be explored to its full potential [2].

The WHO global strategy on digital health 2020–2025 aims to assist countries in adopting suitable digital technologies aligned with their health priorities, advancing towards universal health coverage and the health-related Sustainable Development Goals. Recognizing the transformative potential of information and communications technologies, the global health community acknowledges their strategic and innovative use as essential enablers [2]. Disruptive technologies, such as the Internet of Things, remote monitoring, blockchain, and smart wearables, have demonstrated their value in improving health outcomes and advancing healthcare support [2]. These innovations contribute by fostering knowledge acquisition, developing new skills, and enhancing the competence of healthcare professionals [2].

In the digital health field, several specific challenges hinder progress. One significant issue is the sensitivity of health data, which raises privacy concerns [3]. The digitization process amplifies the vulnerability of data to breaches, unauthorized access, and misuse, highlighting the critical need for robust data protection measures [4]. Governments face difficulties in managing and protecting this data due to the evolving nature of cyber threats and the complexity of implementing comprehensive security frameworks [5].

The absence of evidence-based standards in digital health also presents a significant issue. This gap makes it challenging to evaluate digital health interventions’ effectiveness, safety, and quality, leading to uncertainty among healthcare providers and patients [3].

Data governance is also a crucial issue in healthcare. Effective data governance ensures health data’s integrity, accuracy, and appropriate use [3]. However, many healthcare organizations struggle to establish clear policies and procedures for data management, leading to fragmented data systems that hinder interoperability and seamless data exchange among healthcare providers and institutions [3].

Furthermore, there is a significant lack of evidence regarding the impact of digital health strategies on health outcomes, cost-effectiveness, and system efficiency [6]. The absence of robust evidence makes it challenging to justify investments in digital health technologies and to persuade stakeholders of their value. As a result, there is a reluctance to adopt these innovations widely, thus slowing down the potential benefits digital health can bring to healthcare systems [3].

Addressing these challenges is crucial to realizing the full potential of digital health innovations in improving healthcare delivery and outcomes. The use of digital technologies should consider the sensitive nature of health information and safeguard security and digital privacy as a human right [7]. Therefore, the development of digital health should prioritize the principles of confidentiality, accessibility, scalability, replicability, interoperability, privacy, security, and transparency [2].

These characteristics align with those of blockchain technology. Blockchain was initially introduced in the context of Bitcoin [8] but has already showcased its potential in various other domains where trust plays a pivotal role [9,10]. Blockchain is a form of Distributed Ledger Technology (DLT) characterized by several key attributes. It operates distributed across various computers, referred to as peers, which maintain copies of the data, known as the ledger. This distribution ensures resilience to power or communication outages, as the data is accessible from multiple geographical locations. To record data, consensus among peers is mandatory, facilitated by specialized algorithms, date-stamping, and cryptographic mechanisms, ensuring its authenticity [8]. Furthermore, the blockchain follows an append-only principle, preventing the alteration or deletion of existing data [11]. New data is accumulated in a block linked to preceding blocks, giving rise to the term blockchain. This immutability renders it suitable for preserving historical data and instills trust by preventing unauthorized data tampering [8,9].

Since its inception, some blockchains have integrated new features, such as executing smart contracts. Smart contracts are Turing-complete computer programs stored on the blockchain that execute autonomously based on pre-established conditions set by their code, which cannot be changed once deployed [12]. They provide a decentralized environment by ensuring that pre-established rules, encoded within the contract, are automatically executed. This feature allows participants to engage in fair and transparent exchanges without relying on a trusted third party. The decentralized nature of smart contracts instills confidence in participants, as they can be certain that the agreed-upon rules will be faithfully executed, fostering trust in the exchange process [13].

One of the use cases of Smart contracts deployed on blockchains is the tokenization of assets available in the physical or virtual worlds [14]. Tokenization is the process of transforming data or assets into a random digitized sequence of characters, also known as a token. Tokens act as a representation of the original data or asset on the blockchain [14].

Tokens can be broadly categorized based on their fungibility into two main types: fungible and non-fungible tokens (NFTs). Fungible tokens are indistinguishable and can be exchanged on a like-for-like basis with other tokens within the same category (e.g., it is irrelevant which banknote one holds) [15]. Non-fungible tokens possess unique identities, are indivisible, and may hold different values or characteristics compared to other tokens (e.g., two paintings). Unlike fungible tokens, NFTs cannot be interchanged on an equivalent basis with another NFT. NFTs find applications in representing the existence and ownership of digital assets, ensuring their authenticity, traceability, and facilitating ownership transfer through trading. Examples of assets associated with NFTs include collectibles or certificates [14].

To enhance the composability of smart contracts, tokens adhere to specific guidelines set by their respective blockchains, with the Ethereum Request for Comments (ERCs) being a widely recognized framework. ERCs outline fundamental functionalities and offer directives for tokens to operate seamlessly within the Ethereum network. In this context, fungible tokens adhere to the ERC-20 standard [16], characterized by its major feature being tradable against any other compatible token on crypto exchanges. Non-fungible tokens (NFTs), on the other hand, conform to the ERC-721 standard [17], specifically employed for tasks such as image storage, authentication, ownership, and digital rights implementation [16,17]. NFTs can also be dynamic, composable, and soulbound. Dynamic NFTs are tokens capable of changing over time, such as virtual art modified by the owner. Changes in a dynamic NFT often refer to changes in the NFT’s metadata triggered by a smart contract [18]. Composable NFTs (ERC-998) can be strategically combined, either in a top-down or bottom-up approach, with other NFTs to generate new, updated, or more representative tokens [19]. On the other hand, soulbound NFTs are tokens intricately tied to a specific user or account, rendering them non-transferable to any other user, making them ideal for applications like individual credentials [20]. To enhance the functionality and efficiency of the ERC-20 [16] and ERC-721 standards [17], the ERC-1155 multi-token standard [21] is also available. It allows for managing and representing multiple tokens within a single smart contract, facilitating a more versatile and streamlined approach to tokenization [21].

In recent years, NFTs have gathered remarkable attention from industrial and scientific communities [13]. Initially recognized for transforming the art world with the introduction of digital collectables, NFTs have become versatile tools with applications spanning various industries. In the collectibles sector, NFTs authenticate rare digital items like trading cards and virtual pets, like the popular CryptoKitties [22]. In digital art, NFTs have resolved issues of scarcity and duplication, offering artists a means to sell unique digital pieces and establish digital ownership. Beyond art, NFTs have found a significant presence in gaming, music, real estate, and identity verification [22,23,24,25]. In gaming, they represent unique in-game assets with aspects of art, collectability, and utility. In the music industry, NFTs offer a new model for fair royalty distribution, allowing artists to sell music and royalties. Real estate sees potential with NFTs tokenizing property deeds for transparent and efficient transactions. NFTs also play a role in identity verification; NFTs tokenize individuals’ identities on the blockchain, offering a secure and decentralized proof of identity [22].

The healthcare sector also recognized the promise of NFTs in addressing challenges in combating the counterfeiting of medical devices, supply chain management, electronic health records, or digital twins [26]. Leveraging blockchain technology, NFTs enable the tracing, tracking, authentication, and validation of medical devices in the digital market [27]. Other studies explore the application of blockchain and NFTs in logistics management, supply chain operations, and drug traceability within the healthcare sector [28,29].

As the technology continues to mature, NFTs are expected to bring about further innovative changes in how various industries navigate the digital landscape. While there have been numerous reports on the use cases of NFTs in various contexts and a noticeable interest from healthcare stakeholders [30,31], there are no systematic-level studies on their application in the healthcare sector. Therefore, there is a need for a systematic-level study of non-fungible tokens in healthcare to answer the following research question (RQ):

RQ: What applications can non-fungible tokens (NFT) have in the healthcare sector?

To answer this RQ, this paper aims to provide a comprehensive and systematic examination of the use cases of NFTs in healthcare, analyzing their specific applications, purposes, and associated research challenges.

## 2. Materials and Methods

This systematic review followed the Preferred Reporting Items for Systematic Reviews (PRISMA) guidelines (http://www.prisma-statement.org, accessed on 28 December 2023).

Guidelines for systematic reviews described by Webster and Watson [32] were also used to identify relevant scientific literature rigorously and transparently and ensure consistency in decision-making.

### 2.1. Search Strategy

Relevant publications were searched in a diversified set of electronic databases: MEDLINE (accessed through PubMed) to cover biomedical and life sciences papers, the ACM Digital Library, IEEE Xplore to account for papers focused on engineering and technology, and Web of Science, EBSCO, and Scopus due to their broad coverage.

Keywords were derived from Medical Subject Headings (MeSH terms) and the keywords found in relevant papers, synonyms, and self-determined search terms. They were organized into groups for systematic classification. During the initial assessment, it was observed that the search term “NFT” retrieved articles on topics unrelated to non-fungible tokens. To mitigate this issue, a keyword exclusion group was added. The resulting groups and search strings are detailed in Table 1.

Different combinations of keywords and MeSH terms were used to search the electronic databases, including search filters and minor variations to account for the different user interfaces of each database. A structured overview of the search process is provided in Table 2.

### 2.2. Eligibility Criteria

This study focuses exclusively on research articles on the applications of non-fungible tokens in the healthcare sector. Therefore, we aimed to include qualitative, quantitative, and mixed studies delineating NFT applications across diverse healthcare stages, ranging from manufacturing and distribution to delivering health services to a patient. Only articles published after 2014 were considered, the year when Kevin McCoy created the first NFT [33]. The search was carried out in November 2023.

### 2.3. Selection Process

Rayyan QCRI [34] managed the references and facilitated the selection process. Two reviewers used this tool to independently evaluate and classify articles individually, according to the title and abstract, into one of three categories: “exclude”; “maybe”; and “include”. Following the title and abstract screening, articles marked as “maybe” and “include” underwent a comprehensive evaluation. Those meeting the inclusion criteria were retained for further analysis and data extraction. Disagreements concerning eligibility among the reviewers were resolved through Zoom meetings, during which the rationales for the assigned classifications were presented and discussed.

### 2.4. Data Extraction

Studies meeting the inclusion criteria underwent data extraction. Data was collected in a structured format and documented in an independent Microsoft Excel spreadsheet. The data were recorded as follows: Author(date), author’s field of work, type of study, study overview, category and field of application, NFT use case, and NFT purpose.

### 2.5. Quality Assessment

The quality assessment methodology was selected following the Information Systems (IS) field. Gradually, design research gained more emphasis compared to empirical and critical research [35].

Design research evolved in identifying new purposeful artefacts/problems, the objectives of the solution/proposal, the methodology applied, the development, the evaluation, and findings communication.

Each article was assessed across four categories of DSR limitations: (a) Input knowledge and technology, (b) Research process, (c) Resulting artifact, and (d) Design knowledge. In total, 19 limitations were evaluated and scored/color graded according to the risk of bias: 0/Red—High risk of bias; 1/Yellow—Unclear risk of bias; and 2/GreenLow risk of bias.

According to the DSR methodology, the issue of “knowledge and technology inputs” is managed by the presence of (1) limited data or their poor quality (L1), which is mitigated by the possibility of collecting reliable data; (2) an insufficient number of previous studies (L2), which limits the potential comparison of results; (3) sample size (L3); and (4) the novelty or shortcoming of the technology (L4), which may create problems that need to be reconsidered in the future.

The theme of the “research process” includes (1) the setting (L5), taking into account the specific nature of the organization; (2) the participants (L6), providing a heterogeneous sample for an integrated view of the problem; (3) the method (L7), the lack of evaluation interfering with the correct implementation of the research; and (4) the difficulties of control, (L8) with difficulties in discussing the results.

On the subject of “results artifact”: (1) simplifications (L9)—considering the advantages of small-scale versus large-scale testing; (2) measurement of bias (L10)—trying to include experts in the early stages of design and development, and explain the empower impact; (3) control experiments (L11)—consider complementary deployment, laboratory versus academic, in their suitability in the problem/solution relationship; (4) real situation/prototype (L12)—if the artefact under development has not been used in a real situation, it must support the result in the projectability of the research considering situations and behavior in social and technical; (4) limited performance (L13); and (5) requirements to use the artifact (L14), to identify the real users’ needs (problem definition) [30].

On the subject of “design knowledge”: (1) uncertainty in future events/time-related constraints/risks (L15), to develop artifacts for emerging situations and identify potential scenarios and implications; (2) outcome not compared to alternatives (L16), to clearly explain the shortcomings of alternative approaches or competing artifacts; scope (L17) to address the artefact adequately, so it is a delimitation and an opportunity for future work; (3) theoretical limitations (L18); and (4) generalizability and transferability (L19).

## 3. Results

The electronic search resulted in 1902 articles, of which 505 were duplicates. Following title and abstract screening, 1344 articles were excluded, and the remaining 53 articles underwent full-text assessment. During the title and abstract screening stage, several articles were initially retrieved for covering “NFT” acronyms unrelated to non-fungible tokens, including terms such as nitrofurantoin, nafithromycin, nutrition facts table, no-flow time, neurofeedback therapy, nutrient film technique, nonfunctioning adrenal tumour, neurofibrillary tangle, nasal feeding tube, neuropsychological tests, natural frequency technology^®^, nerve flossing technique, non-fast-track, no feeding tube, nonfluoride toothpaste, newly formed tissues, neural field theory, normal fat tolerance, natural frequency tree, nano-fiber tablets, no further therapy, non-filling thrombus, nifurtimox, nitrogen footprint tool, non-face touching, nonlinear fourier transform, nonfellowship trained, near freezing temperature, and near-field transducer. To enhance search efficiency, future studies may use these terms for exclusion besides the ones originally stated in the G3 line of Table 1.

After full-text assessment, 39 articles were deemed out of scope, and 14 remained for further analysis and data extraction. An additional article was identified through citation-searching in relevant papers, bringing the total to 15 articles for analysis and data extraction.

**Figure 1:**
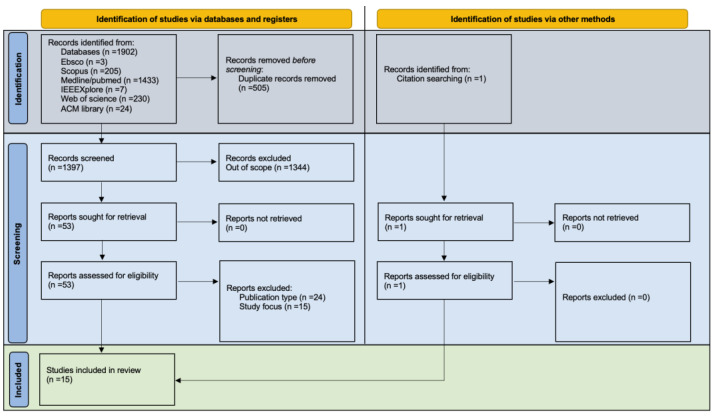
PRISMA flow diagram of the performed systematic literature review.

The PRISMA flow diagram in Figure 1 summarizes the entire article searching, filtering, and selection process. 

The final stage resulted in 15 peer-reviewed articles from countries such as the USA, UAE, UK, India, Tunisia, Saudi Arabia, Vietnam, Canada, China, Iran, Iraq, and Italy.

The final 15 articles underwent data extraction, and a comprehensive summary is presented in Table 3.

**Figure 2:**
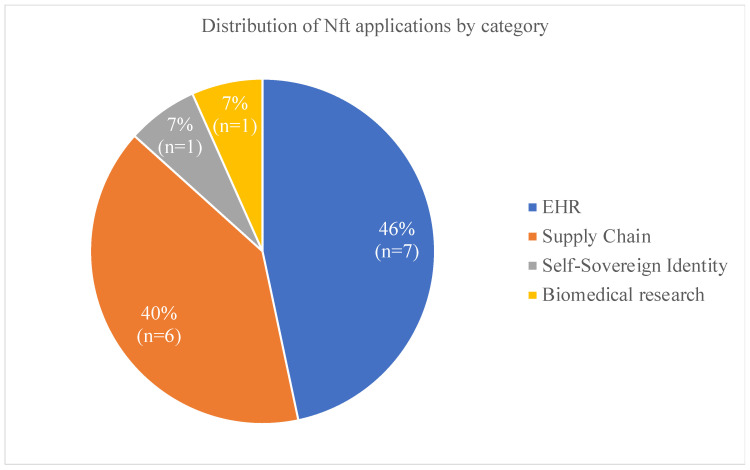
Frequency of NFT use by category.

The type of study and the overview are reported by the authors or inferred from the study details if not explicit. The articles address applications of NFTs in medical devices, digital pathology exams, intelligent diagnosis/machine learning, pharmaceuticals, medical records management, healthcare products, patient consent, medical waste management, research funding, identity management, and patient-generated health data management. Figure 2 summarizes the NFT field of application according to their broader healthcare categories.

**Figure 3:**
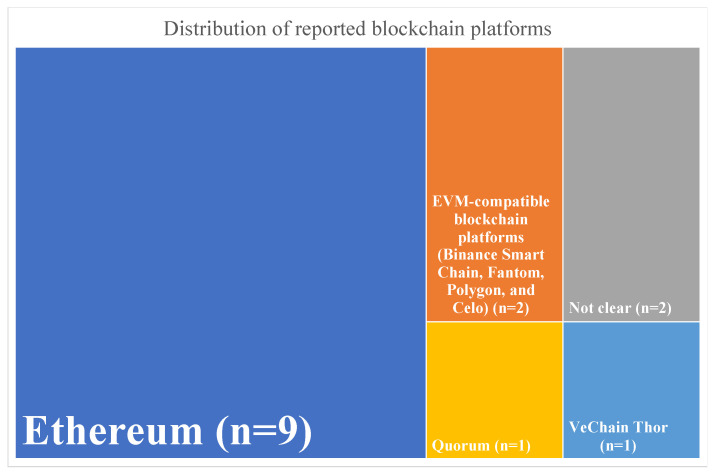
Distribution of included articles by blockchain platform used.

Considering the concern in the included articles regarding data storage, a brief description was provided whenever explicit in the study details, differentiating between on-chain (on the blockchain) and off-chain (off the blockchain) storage. The identification of the blockchain platform used is presented, and the results are summarized in Figure 3.

In instances where more than one type of NFT was employed in a single study, their objectives were clarified based on their smart contract functionality. Lastly, a concise conclusion regarding NFT implementation was included in each article.

### Methodological Quality Assessment

**Figure 4:**
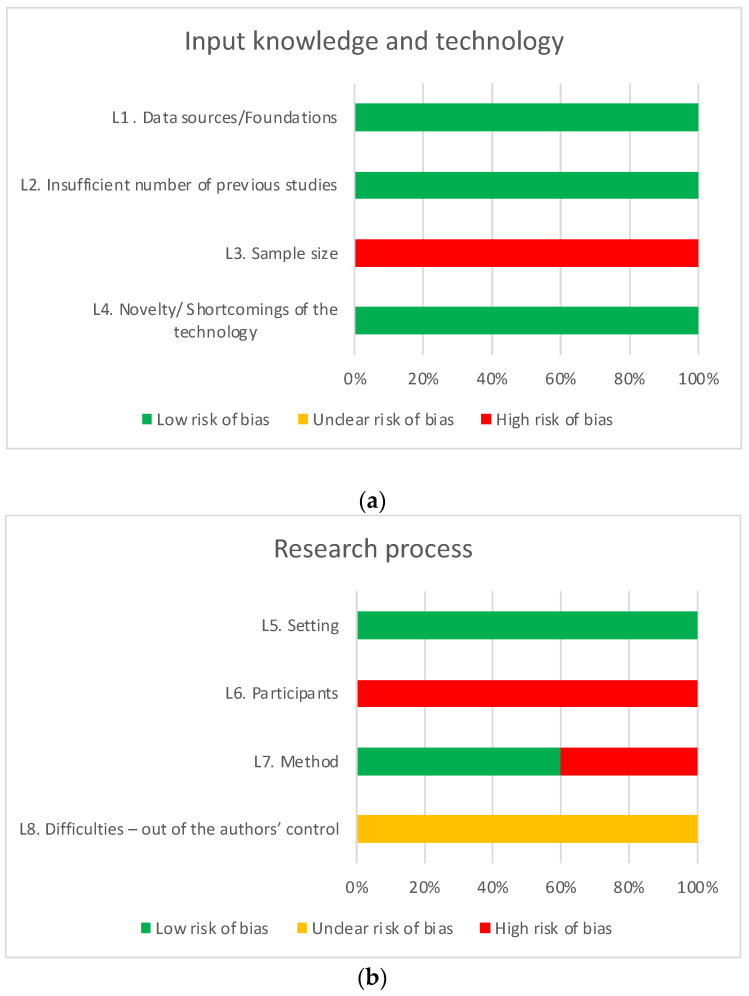

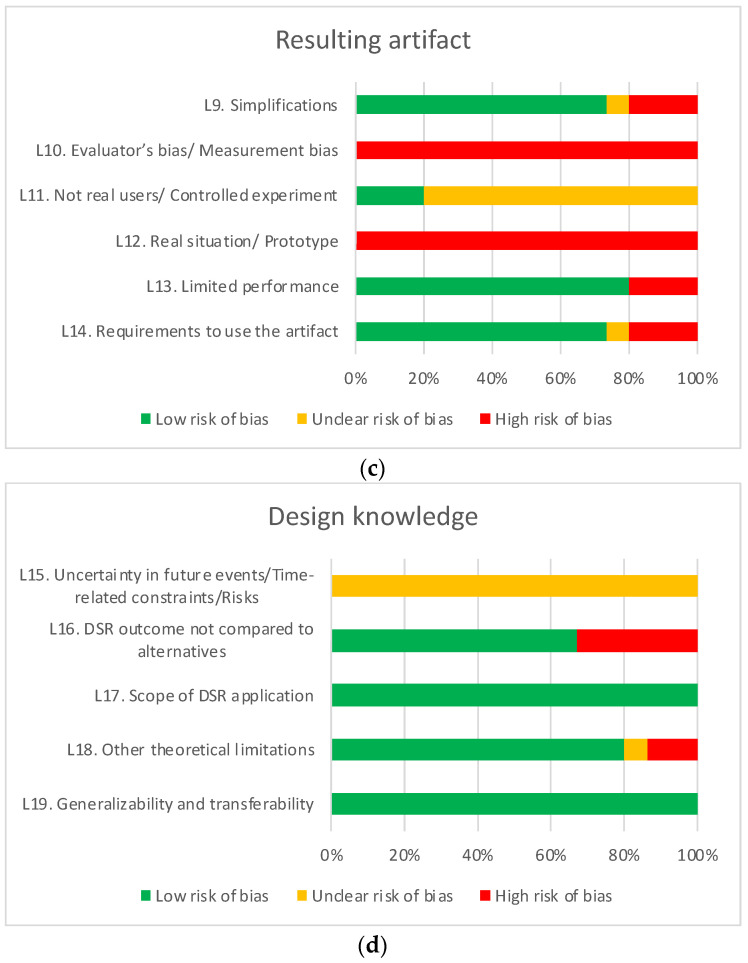
(**a**). Summary of input knowledge and technology quality assessment results. (**b**) Summary of research process quality assessment results. (**c**) Summary of resulting artifact quality assessment results. (**d**) Summary of design knowledge quality assessment results.

The results of the methodological quality assessment are presented in Table 4, and each DSR limitations category is schematically represented in Figure 4a–d.

The category Resulting Artifact showed a higher prevalence of a high risk of bias or unclear risk of bias. There is a noticeable high risk of bias common to all articles in the limitations L3, sample size; L6, participants; L10, evaluator’s bias/measurement bias; and L12, real situation/prototype. This observation is consistent with the fact that all included articles are in the proof of concept or system design phase, having not yet undergone an experimental phase in a real environment with real participants and external or expert evaluation. For the same reason, all included articles present an unclear risk of bias regarding external difficulties and future challenges, as presented in L8, Difficulties–out of the authors’ control, and L15, uncertainty in future events/time-related constraints/risks.

Regarding limitations related to the research process methodology, L7, method, 40% of the included articles were classified with a high risk of bias for not providing clear information about the blockchain used or data storage in the proposed system.

With the exception of limitation L15, the category Design Knowledge was generally classified with a low risk of bias. The identified shortcomings relate to the lack of comparison with currently implemented or similar alternative systems to the proposed system (L16) and the presentation of theoretical limitations where possible challenges of using NFTs and blockchain or implementing these systems in healthcare systems were not considered (L18).

## 4. Discussion

In the global health community, the strategic application of Information and Communication Technologies (ICT) in the health sector is widely acknowledged as a crucial facilitator in pursuing universal health coverage and enhancing overall health and well-being [2]. According to the World Health Organization (WHO) in its Global Strategy on Digital Health 2020–2025 [2], the implementation of digital health is recognized for its potential to enhance the efficiency and cost-effectiveness of healthcare delivery, paving the way for innovative service delivery models.

To maximize the adoption and added value of digital health initiatives, attention and resources should be directed towards addressing critical areas of concern within the health sector. These include accessibility and support to equitable and universal quality health services; the efficiency and sustainability of health systems in delivering quality, affordable, and equitable care; the strengthening and scalability of health promotion, disease prevention, diagnosis, management, rehabilitation, and palliative care in a system that respects the privacy and security of patient health information [1,2].

Regarding patient health information privacy and security, healthcare organizations have been facing difficulties with personal information leaks [7]. Protecting sensitive medical and personal data found in these organizations demands particular attention to digital privacy, recognized as a fundamental human right by the United Nations [7]. In opinion papers and letters to editors, NFTs have been informally proposed as a transformative solution in optimizing critical processes within the medical field, for example, in hematopoietic stem cell transplants and organ allocation for transplantation [31]. In stem cell transplants, NFTs may offer a streamlined approach by enabling stem cell donor centers to input essential information, such as HLA and ABO blood-typing, as well as transplant availability directly into NFTs. This information becomes publicly accessible for transplant centers, resulting in a real-time efficient inventory management system [51]. Similarly, in the distribution of solid organs, integrating NFTs into the allocation process addresses the complexity and lack of transparency in current practices. NFTs leverage smart contracts, enabling automated and efficient data management, thereby minimizing the risk of errors and miscommunication. This enhanced information accessibility has the potential to drive discoveries, streamline transplant procedures, improve accountability, and instill public trust in the organ transplantation process. Notably, NFTs offer the ability to store sensitive information securely and selectively disclose only the necessary details at the time of transfer, adding layer of security to the process [31].

This systematic review outlines four overarching categories of health NFT implementations: biomedical research, electronic health records (EHR), self-sovereign identity, and supply chain. Within each category, diverse applications are explored, underscoring the acknowledgment of NFTs as a valuable tool with widespread applicability throughout the healthcare sector.

### 4.1. Biomedical Research

#### Research Funding

In biomedical research, Mishra B. and Qi Q. [44] introduce a research funding system designed to democratize the drug discovery process and alleviate drug prices by removing traditional intermediaries that typically separate biomedical researchers from potential patients. The proposed approach uses NFTs to represent distinct tranches in the capital market of research projects, raising funds by selling NFTs based on anticipated future patents, associated risks, and expected returns.

Within this system, investors can build a diversified portfolio of drug development projects. Patients can acquire NFTs for future treatments related to a specific disease either by direct investment or by participating in clinical trials, where they may share their DNA sample or electronic health record (EHR), among other options. Participants in clinical trials may also receive compensation. Scientists, considering the market prices of NFTs for various drug development projects, can focus on projects in high demand from investors. This innovative application of NFTs contributes to increased transparency, reduced market manipulation, and establishing a globalized system. Furthermore, it facilitates scalability and liquidity through a secondary exchange market for the NFTs. By eliminating the need for a central authority to govern the market and other intermediary institutions, the innovative application of NFTs enhances transparency and mitigates the risks of deception and non-transparency typically linked to market manipulation. This decentralized approach ensures more transparent market activities, reducing susceptibility to manipulation. Moreover, it promotes globalization of the system and supports scalability and liquidity through a secondary exchange market for the NFTs.

### 4.2. Electronic Health Records

In the category of EHR, NFTs are proposed for digital pathology exam sharing, patient consent, patient-generated health data management, intelligent diagnosis/machine learning, and medical record management.

#### 4.2.1. Digital Pathology Exams

In the case of digital pathology exam sharing, Subramanian H. and Subramanian S. [37] present a proof of concept for a blockchain-based smart contract system using the NFT standard ERC-721 [17] and the Interplanetary File System (IPFS) for off-chain data storage. This innovative approach decentralizes image storage, authentication, ownership, and digital rights within digital pathology.

The NFTs serve as references and ownership document to health data stored off-chain (IPFS). The data stored on IPFS includes high-resolution image files, modifications made by professionals, and JSON files containing metadata about the scans or the diagnosis provided by the physician. The outcome is a decentralized, secure, and privacy-preserving digital pathology system that effectively addresses data storage and sharing challenges.

This architecture separates the storage of the actual image from its metadata, allowing image transmission with permission granted solely for accessing the metadata file through NFT functions, eliminating the need to move the entire high-resolution image to a different network address. Data record ownership ensures perpetual access to the NFT for the primary owner, the patient while granting access to laboratories, pathologists, and other experts through assigned NFTs. Implementing NFTs for pathology-based image storage holds promising prospects for the widespread adoption of digital pathology, contributing to cost reduction, improved diagnosis speed, and more affordable medical care over time.

#### 4.2.2. Patient Consent

For patient consent, a collaborative effort matching engineering, computer science, and law carried out by Cunningham et al. [42] proposes NFTs as a mechanism for documenting and transmitting patient consent within health data transactions. NFTs offer a solution for data subjects to record digitally signed consent, creating verifiable and auditable consent records. This system enables the transmission of consent from multiple data subjects for research purposes, allowing authorized consumers to access medical data without relying on a trusted third party to validate the legitimacy of these consents. In the context of medical informatics applications, the unique properties offered by NFTs can be particularly valuable in scenarios requiring the exchange and execution of distinct one-time rights. This approach reduces reliance on a single component, enhancing trust in the overall system. The proposed system facilitates the secure transmission of consent from multiple data subjects, empowering legitimate data consumers to apply these consents when obtaining data from medical providers for research purposes.

#### 4.2.3. Patient-Generated Health Data Management

In Subramanian H.’s proposal [48], a decentralized marketplace for patient-generated health data (PGHD) is introduced with the potential to enhance provenance, data accuracy, security, and privacy. The system utilizes non-fungible tokens to represent patient-generated health data records, establishing a decentralized marketplace where various participants, including data creators, sellers, and value-added service providers, can transparently monetize data. PGHD data is uploaded to the Interplanetary File System (IPFS) for storage, and a data content identifier (CID) is generated and stored on the NFT to pinpoint the PGHD data during the exchange and monetization of health data by creators.

On the supply side, patients are responsible for generating data using personal devices and are incentivized to produce high-quality data sets. Buyers on the other side include service providers such as data aggregators, firms offering predictive analytics, and application developers, researchers, or data scientists analyzing and adding value to the data.

To ensure data integrity and quality, the marketplace incorporates several reputation models. Rating and review systems allow users to evaluate each other, with mechanisms to prevent fraudulent reviews, such as weighting expert opinions and manual and third-party content verification. Verification systems confirm user identities and credentials, enhancing trust. Detailed feedback systems inform users about transaction experiences, maintaining high data standards. Trust networks help users build reliable relationships, enabling repeat transactions and the preordering of datasets.

A comprehensive data-correctness strategy includes reputation mechanisms, statistical validation, third-party oracle validation during onboarding, and penalties for fraud detected by third parties. This strategy discourages fraudulent data practices and motivates patients to produce high-quality datasets.

By implementing these reputation models, the marketplace reinforces a reliable supply of accurate data for AI-driven analysis and disease diagnosis, ultimately enhancing the quality of available health data.

#### 4.2.4. Intelligent Diagnosis/Machine Learning

While some studies acknowledge artificial intelligence as a field that can benefit from using non-fungible tokens (NFTs) in image and medical data sharing, Sai et al. [38] introduce a novel framework for intelligent health diagnosis. The approach involves a Federated Learning (FL) framework with a blockchain-based incentive mechanism and an NFT-based marketplace for health data.

NFTs are crucial in this framework by representing patient health records and data copyright. Patients register on the NFT marketplace, providing specific personal details and uploading previous medical data with relevant information. This includes details such as the type of medical report (such as MRI, CT-scan, X-ray, and ECG), labels indicating a specific disease and the date of disease diagnosis. Patients also express their willingness to participate in federated learning model training and receive rewards at the end of each FL training process based on the data they contribute.

FL owners access patients’ health data features on the NFT marketplace and filter and select the NFTs to train their FL models. Upon selection, the FL model owner requests access to these NFTs and pays for data access. A comprehensive incentivization model rewards patients based on attributes such as quantity, quality, relevance of data, and the impact of local model training on global model performance.

The outcome is a privacy-preserving medical record-sharing scheme for intelligent diagnosis in smart healthcare. This approach addresses various issues present in existing intelligent diagnosis frameworks that rely on indirect patient data collection. The combination of federated learning and NFTs contributes to a more secure, privacy-focused paradigm for intelligent health diagnosis.

#### 4.2.5. Medical Records Management

In this systematic review, medical record management emerges as the predominant field of application across all categories, including three studies.

Quy et al. [40] proposed a Medical Test Results Management framework that integrates blockchain, smart contracts, and NFTs. NFTs serve as a repository for personal information and treatment history, encapsulating medical test results. This framework enables seamless data sharing between patients and medical practitioners, encompassing functions against data manipulation such as creation, query, and update on the blockchain platform. By storing personal information and treatment history as NFTs and medical test results in a separate off-chain database, the study aims to achieve a balance between data accessibility and security. Key contributions include a mechanism for sharing medical test results while preserving data privacy and a model for generating certified, NFT-based document sets that encapsulate these results.

Addressing information security concerns, Mohammed M. and Wahab H. [46] propose a system to secure both medical records and Electronic Medical Records (EMR) using NFTs as the storage medium for a single patient’s medical records. The NFTs ensure easy access and guarantee availability, privacy, and security, empowering patients with ownership and control over their data. The study introduces a customized permission blockchain built with secret sharing technology to reduce key management overhead and facilitate easy access from any computer.

In a complementary contribution, Mohammadi S. [50] et al. propose a model for storing and transmitting medical prescriptions using a combination of NFTs and an off-chain centralized data storage system. In this context, NFTs serve as repositories for medical information and prescriptions, ensuring immutability, secure accessibility, data quality, patient consent, efficient record management, and enhanced availability and reliability. The central database stores patients’ medical history, while NFTs act as a cache memory for prescriptions, facilitating faster retrieval for repetitive access. The proposed model leverages NFT technology to certify the immutability of healthcare records on the blockchain, guaranteeing ownership and preventing information disclosure through encryption strategies. This comprehensive approach addresses critical security criteria, such as data integrity, non-repudiation, anonymity, confidentiality, and privacy, outperforming related research in the healthcare domain.

In summary, while all three studies address medical record management with NFTs, they vary in their specific applications, proposed frameworks, and emphasis on different security and privacy considerations. Each study contributes uniquely to the broader goal of enhancing healthcare data management through NFT integration.

### 4.3. Self-Sovereign Identity

#### Identity Management/Medical Records Management

NFTs have been suggested to establish complete control over identity through NFT authentication in medical record management. In this proposal [45], NFTs function as unique smart contracts exclusively for the patient’s use, serving as a unified identifier across various healthcare facilities. This approach empowers patients with ownership and control over their identities, allowing them to determine how and with whom their personal health information and medical history are shared.

In this system, sensitive data and medical records undergo encryption and are stored and exchanged through a distributed file system (IPFS). Only hashes for the encrypted data are retained in the NFTs. Implementing a Self-Sovereign Identity-enabled patient tokenization system results in a transparent and efficient data-sharing system that allows patients to authenticate themselves without requiring an intermediary party, minimizing the risk of protected health information breaches. By leveraging blockchain’s distinctive features, healthcare facilities can anonymously link local patients’ identifiers to NFTs for future health information exchange requests. Smart contracts and robust security properties ensure regulatory compliance throughout the processes.

Patients can always audit the records’ access history by tracking the blockchain history, providing an additional layer of transparency and accountability to the entire system.

### 4.4. Supply Chain

In the supply chain category, NFTs are proposed for managing and distributing medical devices, including the refurbishment process, pharmaceuticals, and healthcare products in general. Medical waste management has also been proposed.

#### 4.4.1. Medical Devices/Medical Devices (Refurbished)

In the two studies focused on the management of medical devices, the common thread is the utilization of non-fungible tokens (NFTs) as digital representations of physical medical devices (digital twins), with both studies authored by S. A. Gebreab [36,47]. The first study [36] proposes an NFT-based traceability and management system for medical devices in general, while the second study [47] narrows its focus to refurbished medical devices. Both articles employ an Interplanetary File System (IPFS) for off-chain data storage, housing the digital representation of the physical device, NFT metadata, images, and any pertinent details related to the medical device. On-chain, only the hash of the file, along with the token ID and Ethereum address of the owner, is stored in the NFT. The use of NFTs harnesses the intrinsic features of the blockchain, ensuring immutable and tamper-proof provenance data, integrity, reliability, non-repudiation, and transparency. This approach enhances traceability and authenticates medical devices, establishing a secure, transparent, and verifiable record of the distribution and refurbishment processes to ensure the safety and quality of these devices.

In Gebreab et al., 2022 [36], focusing on a general medical device system, ERC-721 NFTs serve for device verification and ownership throughout the distribution process. In contrast, Gebreab et al. 2023 [47], centered on a refurbished medical device system, introduces ERC-998 NFTs, which are composable and dynamic. In this hierarchy, medical devices and replacement parts (represented as ERC-721 tokens) are structured as parent-child NFTs. Any changes or modifications made to the refurbished device are virtually reflected through the dynamic nature of these implemented tokens. Notably, non-transferable NFTs, also known as Soulbound Tokens, are introduced to represent refurbishment certificates. These certificates are permanently linked to the corresponding medical device NFT, preventing any attempts to falsely sell the device as new once the certificate is issued. This innovative use of NFTs ensures the integrity of the refurbishment process and enhances the overall transparency and trustworthiness of the medical device lifecycle.

#### 4.4.2. Pharmaceuticals and Healthcare Products

In studies focused on pharmaceuticals and healthcare products, three distinct systems were proposed: Turki et al. [39] introduced an NFT-IoT Pharma chain, an IoT-based pharmaceutical supply chain integrating blockchain and NFTs. NFTs were used as digital twins for medicine lots (Lot NFT), medicine orders (Order NFT), and distribution vehicle addresses (Vehicle NFT). The objective was to ensure secure and transparent product traceability, providing a holistic view of medicine lots, including details such as the current owner, position, environmental shipment conditions, and certifications. The data storage utilized IPFS to store license and actual data, generating a hash for retrieval, with the hash stored in the blockchain. IPFS off-chain storage maintained various NFTs, preserving sensitive information privacy. This Ethereum-based system facilitated easy tracing, ownership proof, and potential royalty earnings for certain actors during successful trades on tokenized medicine lots.

Musamih et al. [41] proposed NFTs for Product Management, Digital Certification, Trading, and Delivery in the Healthcare Supply Chain. NFTs served as digital twins for healthcare products, ensuring ownership, provenance, and authenticity. The system aimed to enhance overall coordination within the healthcare supply chain by directly linking product ownership to the controlling entity. Similar to previous studies, data storage utilized off-chain IPFS for metadata, avoiding the storage of large-sized data on the blockchain. This NFT-based approach is aimed at decentralized, transparent, secure, reliable, auditable, and trustworthy data ownership and provenance. Users could tokenize healthcare products for easy trading and tracing with integrated data analytics tools linked to a trusted, secure, reliable, and transparent database, enabling independent analyses without reliance on third parties.

Chiacchio F. et al. [49] proposed a decentralized solution based on NFTs to improve the track and trace capability of the standard serialization process in the pharmaceutical industry. NFTs acted as digital twins for serialized pharmaceutical items, enhancing track and trace processes, communication among supply chain stakeholders, and building consumer trust. The study utilized the VeChain blockchain platform instead of Ethereum, and data storage followed the principle of separating data, using a centralized database rather than IPFS, while maintaining the same data privacy practices. This approach reinforced the generic serialization technology used by pharmaceutical manufacturers, ensuring seamless integration without modifying the standard serialization process.

### 4.5. Medical Waste Management

In medical waste management, non-fungible tokens (NFTs) are a reward/punishment system designed for individuals and institutions. The objective is to cultivate heightened awareness and conscientiousness toward effective waste classification within healthcare settings. These NFTs encapsulate pertinent evidence and information about an individual’s or organization’s compliance or violation and waste segregation behavior. The resulting model, crafted to enhance waste classification and management practices in Vietnam, seamlessly integrates blockchain technology, smart contracts, and NFTs. The overarching aim is to instigate a cultural shift towards improved waste management practices within healthcare facilities on both an individual and collective level [43].

### 4.6. Technical and Ethical Considerations

In medical informatics applications, non-fungible tokens (NFTs) emerge as a mechanism that eliminates the need for centralized sources of trust within health informatics architectures. Moving forward, NFTs are poised to play a substantial role in advancing eHealth applications.

This review underscores the versatility of NFT-based solutions, showcasing their applicability across various stages of healthcare.

While the proposed systems could theoretically be implemented using traditional informatics architectures, the unique attributes of a blockchain-based NFT solution set it apart. The capability to transfer rights privately, securely, and freely, independent of any centralized entity’s knowledge and control, distinguishes this solution, offering flexibility and security unmatched by traditional approaches.

Despite their advantages, implementing new technologies can impact users and create new problems. Overcoming the complexity of adopting blockchain in healthcare remains a significant challenge, particularly for health professionals accustomed to traditional care methods [52,53,54]. Many users may not fully understand the conditions of use when they agree to share their health data, leading to potential exploitation or misuse of their information [3]. Resistance to change underscores the need for comprehensive training sessions and workshops to showcase blockchain technology’s and NFTs’ advantages and functionalities [52]. Additional technical support may be necessary during the transition period to facilitate implementing and integrating these innovative systems. Managing overhead costs, including expenses for platform development, system assessment, and ongoing maintenance, is also a key concern [52]. In essence, while blockchain technology offers significant potential for improvement in the healthcare sector by improving data security, efficiency, and transparency, its implementation is intricate. Challenges include technical barriers, the necessity for comprehensive training, additional resource requirements, and ethical considerations. Overcoming these challenges demands a multifaceted approach involving technological innovation, stakeholder engagement, and ethical oversight to realize the benefits of blockchain NFT-based healthcare solutions without compromising healthcare service quality and integrity.

Some existing blockchains incur high operational costs due to the need for expensive hardware and substantial energy consumption. However, this is gradually changing. For instance, Ethereum, one of the most popular blockchain networks, is transitioning from a Proof-of-Work to a more energy-efficient Proof-of-Stake consensus mechanism as part of its ongoing upgrades. This strategic shift aims to enhance the network’s scalability, security, and sustainability, thereby encouraging broader adoption [55]. When considering potential solutions, it is important to carefully evaluate temporal volatility’s impact on transaction finances. In NFT-based solutions, transactions involving transaction fees are recorded on the blockchain, such as NFT creation, ownership transfers, or information updates. Unlike traditional fees solely dependent on the amount of information, these costs are dynamic and influenced by factors like network states and supply and demand dynamics. Additionally, distinct attributes of each blockchain platform, including consensus mechanisms, block time, and network size, significantly contribute to transaction costs. Therefore, when choosing a blockchain platform for deployment, a thorough evaluation of these platform-specific variables is crucial [40].

Our findings reveal that 73% of the studies opted for Ethereum or Ethereum Virtual Machine (EVM)-compatible platforms. EVM-compatible platforms in this review include Binance Smart Chain, Fantom, Polygon, and Celo. EVM-compatible platforms refer to blockchain networks that can deploy Ethereum-based smart contracts or applications without major changes, improving interoperability and maintaining a high level of security [56].

After analyzing and comparing costs across EVM-compatible platforms, Quy et al. [40] concluded that BNB Smart Chain was the most expensive platform. In contrast, Polygon, Celo, and Fantom offer significantly more cost-effective alternatives. Fantom and Polygon present remarkably low transaction costs, making them potential choices for economically deploying NFTs. However, critical factors beyond cost should be considered, such as platform maturity, ecosystem support, developer experience, security, and user adoption. We argue these factors justify why Ethereum remains the predominant blockchain platform utilized in NFT schemes, offering a secure environment for executing smart contracts despite higher transaction costs.

Using NFTs eliminates the necessity for complex smart contracts, functioning as cache memory to expedite data retrieval for repetitive use, thereby mitigating the risk of higher gas fees and slower transaction times on the blockchain. Consequently, NFT-based solutions are deemed more cost-effective than blockchain-based alternatives that do not support tokenization [36].

In addition to operational costs, a recurring concern in the reviewed articles was the costs associated with storing information, prompting exploring solutions to enhance system efficiency and address scalability challenges linked to blockchain usage.

While a blockchain-based system offers a decentralized approach for storing personal sensitive data within a distributed environment, ensuring user data privacy remains a significant challenge. The potential exposure of user data through data mining and data science technologies poses a notable privacy concern [46].

Data sovereignty and confidentiality hold particular significance in the healthcare sector, given the sensitivity and personal nature of the information involved. The handling of sensitive data and management of electronic health records face significant challenges due to data protection regulations. For instance, the General Data Protection Regulation (GDPR) in the European Union requires careful management of personal data, as individuals have the right to withdraw consent for data processing and the right to be forgotten. Similarly, California’s Consumer Privacy Act, enacted in 2018, gives individuals the right to delete and opt-out of personal information collection. These regulations could potentially present obstacles for applications such as electronic health records because information stored on a blockchain cannot be removed or altered. However, there are technical and legal solutions to tackle these issues. To comply with privacy regulations, there has been a focus on developing customized architectural designs. Previous studies have underscored the importance of pseudonymizing data on the blockchain to ensure that personal data cannot be directly linked to an individual without additional information [57,58]. An example of compliance with GDPR is the three-level architecture of Germany’s Federal Office for Migration and Refugees Blockchain Solution, as presented by Rieger et al. [57]. This solution involves separating digital identity from blockchain transactions. Additionally, these hybrid solutions that store only minimal data on the blockchain can help reduce costs and alleviate concerns about energy consumption and environmental impact [59]. In this sense, NFTs could play a crucial role by exclusively revealing the wallet address of their owner and maintaining the anonymity of the actual user’s identity. Sensitive information is securely stored off-chain, with only the unique identifier stored on-chain, ensuring that confidential details are never exposed to external users scanning the Blockchain. Stakeholders’ identities are protected by their Ethereum addresses, ensuring confidentiality in healthcare transactions. This measure prevents malicious users from mapping specific details related to an individual, reinforcing privacy and security in healthcare data management while supporting data protection regulation.

From analyzing the results of this systematic review, five lacked clarities on data storage management, while the remaining ten advocated for separating information storage from the actual NFTs. In these systems, medical histories, high-resolution images, or processed data are stored in off-chain databases, detached from the blockchain, with only an identifier stored in the NFT metadata for future access.

Storing the encrypted unique identifier on the blockchain as part of the NFT proves to be more cost-effective than directly storing the data on the blockchain [38]. By adopting a strategy of off-chain storage for larger files coupled with NFTs for health data tracking, these systems preserve advantages of blockchain, such as transparency, while fostering scalability for larger datasets, consequently reducing transaction costs [50].

Regarding off-chain data storage, proposed alternatives involve centralized databases and decentralized protocols, such as the InterPlanetary File System (IPFS), both deemed less expensive than direct blockchain storage. [38,50]. However, the choice between them entails trade-offs. While centralized databases offer cost-effectiveness, they introduce vulnerability to security breaches and downtime due to a reliance on a single centralized point of control. Conversely, IPFS leverages cryptographic hashes for file verification, ensuring data integrity and authenticity, thereby enabling verifiable and resilient decentralized storage with no single point of control or failure. In NFT-based solutions this works by storing a unique identifier, Content IDentifier (CID), in the NFT smart contract. IPFS mitigates data silos typically associated with centralized servers, rendering it more resilient than traditional centralized systems. Moreover, it safeguards data sovereignty, thereby holding the potential to revolutionize the existing paradigm towards a fully decentralized system [60].

In addition to privacy considerations, several technical aspects make NFT-based solutions highly relevant in the healthcare sector [36,37,38,39].

Data Integrity: Past transactions and ownership transfers are securely recorded on the blockchain, providing provenance data and a tamper-proof history of lifetime health data or medical devices and pharmaceuticals supply chain.

Availability: Smart contracts record all logs and data within the blockchain and remain accessible to all system users and stakeholders, ensuring dependability, reliability, and usefulness. Even in a malicious or denial-of-service attack, all transactions and data in the blockchain remain accessible.

Non-repudiation: Stakeholders’ addresses are registered, and every function call made by an entity is recorded in immutable transaction logs, preventing any denial of actions. In the blockchain, entities cannot repudiate an action or transaction, as everything is documented in tamper-proof logs.

Authentication and Authorization: Smart contracts ensure that functions can only be called by authorized entities, blocking access when faced with unauthorized attempts. This aspect becomes particularly pertinent in situations where regulatory compliance is mandatory. Regulatory authorities can be integrated into a system based on their roles.

In specific scenarios outlined in the included articles where regulatory compliance is imperative, smart contracts play a pivotal role in ensuring that only medical devices or health products sanctioned by the regulatory authority meet the criteria for tokenization as NFTs [36]. The user registration process can also be meticulously managed, allowing access exclusively to those who meet stringent regulatory requirements [38].

While there are potential applications of NFTs in healthcare, it is important to address the associated security vulnerabilities. Integrating NFTs in healthcare systems may introduce security vulnerabilities, as healthcare data is highly sensitive and susceptible to cyberattacks.

NFTs are smart contracts that follow standards like ERC-721 and ERC-1155 in Ethereum, making them susceptible to smart contract vulnerabilities. For instance, marketplace platforms that facilitate NFT transactions have their smart contracts, which, if not properly coded, can be exploited, potentially leading to the loss of valuable NFTs. In healthcare, each patient would have a unique NFT representing their data. Still, vulnerabilities like re-entrance could allow malicious users to duplicate these NFTs, complicating the distinction between original and counterfeit tokens [26].

An NFT system combines blockchain, storage, and web applications, making it vulnerable to the weakest component. For example, spoofing can occur if an attacker steals authentication credentials, leading to unauthorized NFT transfers. Tampering can disrupt the integrity of NFT data, particularly if data stored outside the blockchain is altered. Repudiation, where the author of a statement cannot deny sending an NFT, is generally protected by blockchain security, but multiple-signature contracts are recommended to secure transactions further [61].

While the potential of NFTs in healthcare is evident, several challenges require attention. Although smart contracts streamline processes in NFT-based solutions, their effective adoption assumes a level of technological literacy and access to internet-connected devices. Convincing all actors involved in the healthcare systems to embrace this technology may prove challenging due to a resistance to change, lack of awareness, or other factors. Addressing these challenges will demand further research, development, and collaboration with stakeholders in the healthcare industry. Most of the included articles originate from engineering and computer science individuals, with some contributions from health data science, healthcare, law, and management experts. To encourage user adoption and ensure optimal utilization of these technologies, active collaboration among stakeholders from diverse fields is essential for future research endeavors.

Additionally, interoperability plays a crucial role in successfully adopting NFTs in ICT-driven healthcare. Rather than replacing existing health information systems, blockchain and NFT-based solutions aim to optimize current shortcomings. Ensuring interoperability with other innovative technologies is equally paramount. The existing findings already present proposals integrating the Internet of Things (IoT), machine learning, and artificial intelligence, underscoring the importance of a comprehensive and interconnected approach to advance healthcare technologies [38,39].

### 4.7. Limitations

It is essential to understand the potential weakness of the articles in relation to the research design impacting the scope of the study and the results.

Regarding study selection, only peer-reviewed articles from journals accessible in the chosen electronic databases, written in English or Portuguese, were included. Despite attempts to identify additional studies through citation searching, it is possible that articles in other languages or from other sources were missed.

The included articles primarily comprised system proposals and proofs of concept, making it difficult to interpret and synthesize data across all studies. Additionally, the quality of methodology varied among the included articles, and the lack of real-life implementation may impact the overall reliability of the findings.

The limitations identify specific directions for prospective studies. New insights for assessing a standardized methodology and real scenario implementations can mitigate the limitations and guide scientific knowledge within the scope of the review.

## 5. Conclusions

Integrating non-fungible tokens (NFTs) into healthcare applications offers a set of features relevant to the health sector, including decentralization, transparency, immutability, security, traceability, and accountability.

It presents a transformative potential across diverse domains such as biomedical research, electronic health records (EHR), self-sovereign identity, and supply chain management. This systematic review underscores the versatility of NFT-based solutions, demonstrating their relevance throughout the healthcare sector.

NFTs serve as a mechanism for democratizing the drug discovery, fostering transparency, and reducing market inequalities. Electronic health records leverage NFTs in various applications, including digital pathology exam sharing, patient consent, patient-generated health data management, intelligent diagnosis, and medical record management. These applications enhance data security, accessibility, efficiency, and transparent data-sharing practices. Supply chain management leverages NFTs to transform the distribution and traceability of medical devices, pharmaceuticals, and healthcare products while also incentivizing responsible medical waste management.

Technical and ethical considerations emphasize the role of NFTs in addressing challenges associated with centralized sources of trust, enhancing data integrity, availability, non-repudiation, and authentication within health informatics architectures. The review highlights the dynamic nature of transaction costs, platform-specific variables, and data storage methods. Off-chain data storage, using centralized databases or the InterPlanetary File System (IPFS), provides cost-effective solutions while addressing blockchain’s scalability and anonymity challenges.

Despite the evident potential, overcoming current limitations requires concerted research, development, and collaboration efforts. Active engagement with stakeholders across healthcare, regulation, and technology domains is crucial. In conclusion, while NFTs offer transformative solutions in healthcare, addressing challenges and fostering collaboration is imperative for realizing their full potential in the healthcare landscape. Future research efforts should focus on refining and expanding NFT applications for real-life implementations, ensuring ethical considerations, and enhancing user adoption through interdisciplinary collaboration.

## Figures and Tables

**Table 1:** Keyword groups and search strings for electronic database search.

Keyword Group	Keywords	Search String
**G1-NFT**	Non-fungible token Nft Soulbound token	“non-fungible tokens” OR “non fungible tokens” OR “non-fungible token” OR “nonfungible token” OR “nft” OR “nfts” OR “soulbound token” OR “soulbound nft” OR “soulbound tokens”
**G2-Healthcare**	Healthcare Health Medical Medicine Hospital Clinical Dentistry Pharmacy Surgery	“Healthcare” OR “Health” OR “Medical” OR “Medicine” OR “hospital” OR “Pharmacy” OR “Dentistry” OR “Clinical” OR “surgery” OR health*
**G3-Exclusion Terms**	Neurofibrillary tangles Neurofeedback training	“Neurofibrillary tangles” OR “Neurofibrillary tangle” OR “Neurofeedback training“ OR “neuro-feedback training”
**MeSH (M1)**	Delivery of Health Care	
**MeSH (M2)**	Health information technology	
**MeSH (M3)**	Health	
**MeSH (M4)**	Health care sector	

**Table 2:** Search process overview.

Database	Article Fields	Search Query	Filters
**Scopus**	title, abstract, keywords	G1 AND G2 NOT G3	English; Article; since 2014
**Medline**	all files	G1 AND G2 NOT G3	English; since 2014
	all files	G1 AND M1 NOT G3	English; since 2014
	all files	G1 AND M2 NOT G3	English; since 2014
	all files	G1 AND M3 NOT G3	English; since 2014
	all files	G1 AND M4 NOT G3	English; since 2014
**ACM Digital Library**	all	G1 AND G2 AND G3	Journals; since 2014
**IEEE Xplore**	all data	G1 AND G2 AND G3	Journals; Early Access Articles; since 2014
**Web of Science**	all fields	G1 AND G2 AND G3	English; Article; early access; since 2014
**Ebsco**	all fields	G1 AND G2 AND G3	All results

**Table 3:** Summary of the included studies.

Author (Date)	Authors Field of Work	Type of Study	Study Overview	Category	Field of Application	Nft Use Case	NFT Purpose
Gebreab S. A. et al. (2022) [36]	Engineering and Computer Science	System proposal/working prototype	NFT-based traceability and ownership management system for medical devices.	Supply chain	Medical devices	NFT as a digital twin of medical devices	“Augmenting traceability and ensuring authenticity through certification of medical devices”
Subramanian H, Subramanian S. (2022) [37]	Engineering and Computer Science	Proof-of-concept	Digital pathology system that uses blockchain-based smart contracts using the non-fungible token (NFT) standard and the Interplanetary File System for data storage.	EHR	Digital pathology exams	NFT as a reference to metadata stored off-chain (IPFS)	“Implement decentralized image storage, authentication, ownership, and digital rights in the context of digital pathology”
	Healthcare						
Sai S. et al. (2023) [38]	Engineering and Computer Science	System proposal	Federated learning framework for intelligent health diagnosis with a blockchain-based incentive mechanism and NFT-based marketplace.	EHR	Intelligent diagnosis/machine learning	NFT as patient health record	“Allow complete access control over patient’s data. Motivate the patients to store their historical medical data with incentive-mechanisms digitally. Contribute to training intelligent diagnosis models”
	Engineering and Computer Science					NFT marketplace for healthcare data	
Turki M. et al. (2023) [39]	Engineering and Computer Science	System proposal	NFT-IoT Pharma chain, an IoT-based pharmaceutical supply chain that integrates blockchain and NFT.	Supply chain	Pharmaceuticals	NFT as a digital twin of medicine lots (Lot NFT)	“Ensure secure and transparent product traceability. A holistic view of medicine has lots of details, including the current owner, the position, the environmental shipment conditions, and certifications. End-to-end of the medicine history”
						NFT as orders of multiple similar drugs or Lot NFTs (Order NFT)	
						NFT as a distribution vehicle address (vehicle NFT)	
Quy T. et al. (2023) [40]	Engineering and Computer Science	System proposal/blockchain comparison	Medical test results management framework using blockchain, smart contracts, and NFTs.	EHR	Medical records management	NTF as personal information and treatment history	“NFTs for medical test results, enabling the easy sharing of data by patients with medical practitioners”
Musamih A. et al. (2022) [41]	Engineering and Computer Science	System proposal	NFTs for product management, digital certification, trading, and Delivery in the healthcare supply chain/NFT-based solution for the management of products within the healthcare supply chain.	Supply chain	Healthcare products	NFT as a digital twin of healthcare products	“Ensure ownership, provenance and authenticity of healthcare products “
	Healthcare						“Improve the overall coordination of the healthcare supply chain by ensuring that the ownership of healthcare products is directly controlled by the entity that owns them”
Cunningham J. et al. (2022) [42]	Engineering and Computer Science	System proposal	non-fungible tokens as a mechanism for representing patient consent.	EHR	Patient consent	NFTs as the mechanism for recording and transmitting consent between health data consumers and data providers	“Enable data subjects to record signed records of consent. Create verifiable and auditable records of consent from data subjects”
	Law						“Transmitting the consent of multiple data subjects to use their data for research purposes and to allow legitimate consumers of such data to apply these consents to obtain data from medical data providers”
Triet M. N. et al. (2023) [43]	Engineering and Computer Science	System proposal	Foster an increased individual and collective consciousness towards effective waste classification within healthcare settings using blockchain, smart contracts, and NFTs.	Supply chain	Medical waste management	NFT as a reward/punishment system aimed at individuals and institutions	“Encapsulate relevant evidence and information concerning the individual’s or organization’s compliance or violation and waste segregation behavior”
Mishra B, Qi Q (2022) [44]	Engineering and Computer Science	System (business model) proposal	Democratize the drug discovery process and reduce drug prices by cutting the intermediaries that stand between biomedical researchers and future patients.	Biomedical research	Research funding	NFT represents different tranches of the capital market of research projects	“Raise funds by selling NFTs based on research project future patents, expected risk and return”
	Management						“Avoid non-transparency and deception associated with market manipulation”
							“Globalize the system and encourage scaling with liquidity using a secondary exchange market for the NFTs”
Zhuang Y. et al. (2023) [45]	Health Data Science	System proposal	Full control of identity through NFT authentication.	Self-sovereign identity	Identity management	NFT as a unique smart contract only for the patient to use—unified identifier across healthcare facilities	“Patients can own and control their identities, allowing them to decide how and with whom their PHI and medical history are shared, leading to a transparent and efficient data-sharing system that prevents data breaches caused by unauthorized access”
					Medical records management		“Enables patients to authenticate themselves without the need for an intermediary party to minimize the risk of PHI breaches.”
Mohammed M, Wahab H (2023) [46]	Engineering and Computer Science	System proposal	Addressing the information security issues related to the security and privacy of both medical records and EMRs by using NFT, which proves the ownership of the patient on his/her data.	EHR	Medical records management	NFT as a storage for medical records of a single patient	“Ensures easy access and guarantees availability, privacy, and security providing authority to the patient over his data as well as proof of ownership”
Gebreab S.A et al. (2023) [47]	Engineering and Computer Science	System proposal	NFT-based solution for the traceability and management of refurbished medical devices.	Supply chain	Medical devices (refurbished)	NFT as a digital twin of medical devices	“Creates a secure, transparent, and verifiable record of the refurbishment process to ensure the safety and quality of medical devices”
							“Non-transferable NFTs (soulbound) as certificates of refurbishment acts as an effective mechanism for detecting suspect medical devices and instances of fraudulent labeling, thereby increasing buyer confidence and promoting user safety”
Subramanian H. (2023) [48]	Engineering and Computer Science	System proposal	Decentralized marketplace for patient-generated health data that can improve provenance, data accuracy, security, and privacy.	EHR	Patient-generated health data management	NFT as a patient-generated health data record	“Design a decentralized marketplace for PGHD data where different participants, such as data creators, sellers, and value-added service providers, can monetize data transparently”
Chiacchio F et al. (2022) [49]	Engineering and Computer Science	System proposal	Decentralized solution based on non-fungible tokens (NFTs) can improve the standard serialization process’s track and trace capability.	Supply chain	Pharmaceuticals	NFT as a digital twin of serialized pharmaceutical items	“Improve the track and trace capability of the standard serialization process in the pharmaceutical industry”
Mohammadi S, et al. (2023) [50]	Engineering and Computer Science	System proposal	Model for storing and transmitting medical prescriptions using a combination of non- fungible tokens (NFTs) and an off-chain centralized data storage system.	EHR	Medical records management	NFT as information on prescriptions	“Store medical information and prescriptions, certify the information immutability of healthcare records, secure accessibility, data quality, patient consent, efficient record management and better availability and reliability”

**Table 4:** Evaluation of quality assessment of included articles.

	Input Knowledge and Technology				Research Process				Resulting Artifact						Design Knowledge				
Author (Date)	L1. Data Sources/Foundations	L2. Insufficient Number of Previous Studies	L3. Sample Size	L4. Novelty/Shortcomings of the Technology	L5. Setting	L6. Participants	L7. Method	L8. Difficulties–out of the Authors’ Control	L9. Simplifications	L10. Evaluator’s Bias/Measurement Bias	L11. Not Real Users/Controlled Experiment	L12. Real Situation/Prototype	L13. Limited Performance	L14. Requirements to Use the Artifact	L15. Uncertainty in Future Events/Time-Related Constraints/Risks	L16. DSR Outcome not Compared to Alternatives	L17. Scope of DSR Application	L18. Other Theoretical Limitations	L19. Generalizability and Transferability
S. A. Gebreab et al. (2022) [36]	2	2	0	2	2	0	2	1	2	0	1	0	2	0	1	2	2	2	2
Subramanian H, Subramanian S. (2022) [37]	2	2	0	2	2	0	2	1	1	0	1	0	2	2	1	2	2	2	2
Sai S. et al. (2023) [38]	2	2	0	2	2	0	2	1	2	0	2	0	2	2	1	0	2	1	2
Turki M. et al. (2023) [39]	2	2	0	2	2	0	2	1	2	0	1	0	2	2	1	0	2	2	2
Quy T.L. et al. (2023) [40]	2	2	0	2	2	0	0	1	2	0	1	0	2	0	1	2	2	2	2
Musamih A. et al. (2022) [41]	2	2	0	2	2	0	2	1	2	0	1	0	2	2	1	2	2	2	2
Cunningham J. et al. (2022) [42]	2	2	0	2	2	0	0	1	0	0	1	0	0	0	1	0	2	2	2
Triet M.N. et al. (2023) [43]	2	2	0	2	2	0	0	1	2	0	1	0	2	2	1	2	2	2	2
Mishra B, Qi Q (2022) [44]	2	2	0	2	2	0	0	1	0	0	1	0	2	2	1	0	2	0	2
Zhuang Y. et al. (2023) [45]	2	2	0	2	2	0	2	1	2	0	2	0	2	2	1	0	2	2	2
Mohammed M, Wahab H (2023) [46]	2	2	0	2	2	0	0	1	0	0	1	0	0	1	1	2	2	0	2
Gebreab S.A. et al. (2023) [47]	2	2	0	2	2	0	2	1	2	0	1	0	2	2	1	2	2	2	2
Subramanian H. (2023) [48]	2	2	0	2	2	0	2	1	2	0	2	0	2	2	1	2	2	2	2
Chiacchio F. et al. (2022) [49]	2	2	0	2	2	0	0	1	2	0	1	0	0	2	1	2	2	2	2
Mohammadi S, et al. (2023) [50]	2	2	0	2	2	0	2	1	2	0	1	0	2	2	1	2	2	2	2

Green: 2- Low risk of bias; Red: 0- High risk of bias; Orange: 1- Unclear risk of bias.

## Data Availability

The original contributions presented in the study are included in the article, further inquiries can be directed to the corresponding author.
